# An outbreak of influenza A(H1N1)pdm09 virus in a primary school in Vietnam

**DOI:** 10.1186/s13104-015-1551-8

**Published:** 2015-10-15

**Authors:** Tran Nhu Duong, Nguyen Thi Thi Tho, Nguyen Tran Hien, Babatunde Olowokure

**Affiliations:** National Institute of Hygiene and Epidemiology, Hanoi, Vietnam; World Health Organization, Hanoi, Vietnam

**Keywords:** School, Children, Influenza A(H1N1)pdm09, Outbreak, Vietnam

## Abstract

**Background:**

Despite school pupils being at greatest risk during the 2009 influenza pandemic there are limited data on outbreaks of influenza A(H1N1)pdm09 in primary schools in South-East Asia. This prospective cohort study describes an outbreak of influenza A(H1N1)pdm09 in a primary school in rural Vietnam.

**Findings:**

In total 103 cases of influenza-like illness were found among the 407 pupils in the primary school. Ten of these were laboratory confirmed cases of influenza A(H1N1)pdm09 virus. The overall attack rate (AR) was 25 % (103/407), and was highest (41 %) in grade 4 pupils, where the outbreak started. All cases in the outbreak presented with a mild and self-limiting illness, acute respiratory symptoms and fever. Public health interventions to contain the outbreak could explain the lower attack rates in other grades. Ill pupils were asked to stay at home. Oseltamivir was not given to pupils and the school did not close during the outbreak. The last detected case occurred 12 days following identification of the first case.

**Conclusions:**

This is the first report of an outbreak of influenza A(H1N1)pdm09 among pupils in a primary school in Vietnam. High attack rates in Grade 4 pupils suggest shared activities contributed to transmission. The public health response using non-pharmaceutical measures may have played a role in ending the outbreak.

## Background

Worldwide the highest impact of influenza A(H1N1)pdm09 virus was seen in school age children and young adults [1]. There are however few reports regarding outbreaks of influenza A(H1N1)pdm09 in primary schools in rural areas of Southeast Asia.

Vietnam reported its first laboratory confirmed case of influenza A(H1N1)pdm09 on May 31st, 2009, a 23-year old man who returned to Ho Chi Minh City (southern Vietnam) on May 26th, 2009. Following this report the A(H1N1)pdm09 virus spread nationwide and the number of confirmed cases climbed steadily for several months.

Public health authorities were alerted on January 12th, 2010 of potential cases of influenza A(H1N1)pdm09 virus in a primary school in the rural commune of Pha Le. At the time of the outbreak there was no evidence of community transmission of influenza A(H1N1)pdm09 virus, or seasonal influenza activity in the local community. This paper describes the epidemiological and clinical characteristics of the outbreak, and the public health response.

## Methods

### Setting

Pha Le commune in Thuy Nguyen District, Hai Phong is a rural commune with approximately 6700 inhabitants who are mainly farmers and fishermen. The Pha Le primary school is located in the centre of the commune.

From January 12th, 2010, pupils were reported to have fever and other respiratory symptoms. Active case finding was implemented in the school and, clinical and epidemiological information were obtained using a structured questionnaire that was administered using face-to-face interviews with parents. All pupils and teachers were closely followed up daily during the outbreak by a team of 4 district and commune health workers. Those who met the suspect case definition were included in the study. Throat swab samples were taken from the first 16 clinical cases. After collection, specimens were kept at 4–8 °C and then transferred to the National Influenza Centre, National Institute of Hygiene and Epidemiology, Hanoi for testing. Following WHO guidelines, infection was confirmed by virus culture or real time reverse transcription polymerase chain reaction (rRT-PCR) methods. In line with MOH guidelines, this outbreak investigation only required verbal informed consent from parents [2].

### Case definitions

The study was conducted from January 12th to February 8th, 2010 and the case definitions applied followed Ministry of Health (MOH) Vietnam guidelines [2]:

*Suspect case* of influenza A(H1N1)pdm09 virus infection: a person with acute onset of fever (≥38 °C) and at least one acute respiratory symptom (cough or sore throat), no other diagnosis, and close epidemiological link with a confirmed or suspected case in the past 7 days.

*Confirmed case* a person in whom rRT-PCR or viral culture of a nasopharyngeal or throat swab sample was positive for influenza A(H1N1)pdm09 virus.

### Data analysis

Analysis of the epicurve was used to determine the type of outbreak occurring using the distribution of cases over time. The most likely period of exposure was obtained from knowledge of the incubation period (mean and range) of influenza A(H1N1)pdm09 virus, and the dates of illness onset of the first and last cases, and the peak of the outbreak. Attack rates were calculated using the number of confirmed/suspected pupils as the numerator and the total number of pupils in the school/class as the denominator. Statistical significance was determined using the Chi square test, and an alpha level of >0.05 was considered to indicate statistical significance. All reported p values are two-tailed.

## Results

**Fig. 1:**
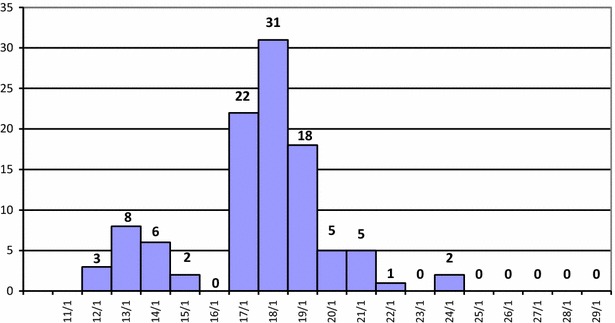
Confirmed and suspected cases of influenza A(H1N1)pdm09 among pupils in a primary school in rural northern Vietnam by date of illness *onset*, January 2010 (n = 103)

The school population comprised 447 persons, 407 pupils and 40 staff members. On 12th January, 2010 three pupils were identified with influenza-like illness (Fig. 1). All three were grade 4 pupils in the same class. The outbreak lasted 13 days (from 12th January, 2010 to 24 January, 2010) and altogether 103 clinical cases (all pupils) were identified. Ten of the first 16 cases tested positive for influenza A(H1N1)pdm09 virus; no further testing was carried out.

The outbreak reached its peak on day 7 (18th January) and no other cases were detected amongst pupils 2 weeks after the last case was reported. The epidemic curve (Fig. 1) shows two successive clusters with the second peak being the highest. This is characteristic of a propagated epidemic, and suggests person-to-person spread with the occurrence of secondary cases. Analysis of the epicurve also suggests that the probable period of exposure for the first cases in the clusters was 1–2 days prior to illness onset, the average incubation period is estimated to have been 1 day. The steep decline in cases may have been as a result of rapid implementation of control measures with a subsequent reduction in transmission to susceptibles.

All age groups in the school were affected (Table 1), and girls appeared slightly more susceptible than boys. Both confirmed and suspected cases had no travel history.

**Table 1:** Pupil attack rates for influenza A(H1N1)pdm09 by grade/age group and sex in a primary school in rural northern Vietnam, January 2010

Category	N (attack rate %)	Attack rate ratio (95 % CI)	P value
Overall attack rate	103/407 (25.3 %)	–	
Attack rate by sex			
Attack rate in boys	49/217 (22.6 %)	Reference	
Attack rate in girls	54/190 (28.4 %)	1.36 (0.85–2.18)	0.18
Attack rate by grade			
Grade 1 (6 years old)	37/106 (34.9 %)	3.30 (1.54–7.15)	0.001
Grade 2 (7 years old)	15/86 (17.4 %)	1.30 (0.54–3.14)	0.53
Grade 3 (8 years old)	13/93 (14.0 %)	Reference	–
Grade 4 (9 years old)	27/66 (40.9 %)	4.26 (1.87–9.85)	<0.001
Grade 5 (10 years old)	11/56 (19.6 %)	1.50 (0.57–3.95)	0.36

All cases in the outbreak presented with a mild and self-limiting illness, acute respiratory symptoms and fever. Sore throat and rhinorrhoea/nasal congestion were reported by the majority of cases (97.1 and 86.4 % of cases, respectively). Cough was observed in only 20 % of cases while fatigue (11.6 %), headache (3.9 %), wheezing (2.9 %) and high fever (≥38 °C) (2.9 %) were less commonly reported. None of the cases reported gastrointestinal symptoms such as vomiting or diarrhoea and no complications or deaths were reported. None of the children had an underlying medical condition, and none had received seasonal influenza vaccination. The median duration between symptom onset and health facility presentation was 3 days (range 0–6 days).

All grades in the school were impacted by the outbreak, and the overall clinical attack rate was 25 % (Table 1). Attack rates by grade ranged from 14 to 41 %. The attack rate was significantly higher (p = 0.0014, using two-tailed Chi square test) among grade 4 pupils (41 %, 27/66), where the initial cases were identified, than among pupils in all other grades (22 %, 76/341).

Public health authorities implemented enhanced surveillance in the school and nearby health facilities. An education campaign was undertaken asking parents to keep ill children at home. Other public health control measures included providing advice on respiratory and hand hygiene, and keeping the school and home environment clean. School (and class) closure and pharmaceutical interventions, such as vaccination and antiviral prophylaxis were not utilised.

## Discussion

This report provides the first description of an outbreak of influenza A(H1N1)pdm09 in a primary school in rural northern Vietnam. Widespread transmission of the virus occurred in the school, only non-pharmaceutical measures were implemented, and the school did not close. The source of infection of the initial cases remains unknown.

Like other diseases transmitted by the respiratory route, the school environment is an ideal setting for institutional transmission and amplification of influenza due to the frequency and intensity of contact between school children, their increased susceptibility to infection, long duration of virus shedding and inability to consistently comply with personal protective measures, such as hand washing [3, 4]. Few studies have specifically reported on outbreaks of influenza A(H1N1)pdm09 in primary schools, and such reports are rare for primary schools in South East Asia. In China an attack rate of 58.3 % was reported following an outbreak of influenza A(H1N1)pdm09 virus in a primary school [5]. The attack rate in China study is substantially higher than that reported from this and other studies [6, 7]. The different in attack rates between the study in China and our study may be due to differences in methodology including the intensity of active case finding, case definitions, and the use of multiple methods to establish a laboratory diagnosis of influenza A(H1N1)pdm09.

This study has some limitations. Parents provided information for their children and this may have influenced the reliability of the data collected. Asymptomatic individuals may also have played a role in transmission, and may have contributed to underreporting of cases. Most persons included in the study did not undergo diagnostic testing, and it is therefore unknown what proportion of these were actually infected with influenza A(H1N1)pdm09 virus. However, 62.5 % of those tested by rRT-PCR were positive, and these results are in keeping with previously published studies where 51–72 % of samples tested positive during an outbreak of A(H1N1)pdm09 [8–10].

The results of this study are also consistent with previous studies that have demonstrated substantial differences in distribution of cases between classes, or year groups [11]. The highest attack rate occurred in the grade with the initial cases, suggesting that the virus initially spread within this grade before transmission to other grades. Further support for this argument is provided by modelling studies where school class was identified as a strong determinant of onward virus transmission [12].

School closure and antiviral prophylaxis are interventions that are often employed, with other measures, in order to reduce virus transmission in schools and communities [6, 13]. However, research on these interventions has largely been reported from well-resourced countries [13]. Further research on the effectiveness of school closure as a pandemic mitigation measure is required in a variety of settings, particularly in rural communities in low income countries where it may be difficult to deliver and access antiviral prophylaxis within 48 h of symptom onset.

## Abbreviations

MOH: Ministry of Health, Vietnam
rRT-PCR: real time reverse transcription polymerase chain reaction
